# Supplementary material for: Pd/Attapulgite Core–Shell Structured Catalytic Combustion Gas Sensor for Highly Sensitive Real-Time Methane Detection
Source: Sensors (Basel). 2025 Aug 10;25(16):4950. doi: 10.3390/s25164950 (PMC12390306; doi:10.3390/s25164950)
Supplement: Supplementary file 1 [file sensors-25-04950-s001.zip › sensors-3763872-supplementary.pdf]

Supplementary Materials of the following article:

Pd/Attapulgite Core–Shell Structured Catalytic Combustion Gas Sensor for Highly Sensitive Real-Time Methane Detection

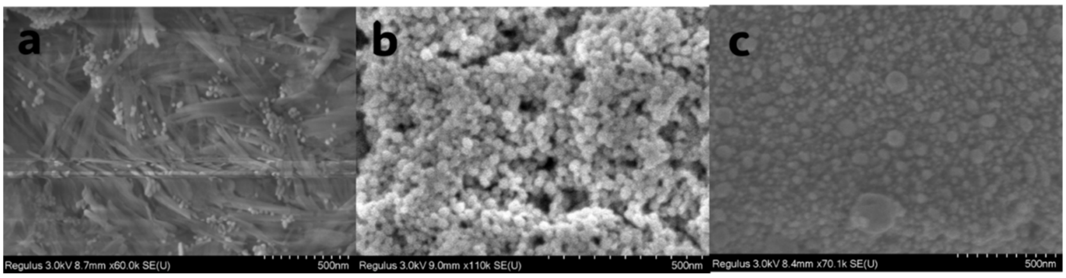

Figure S1. Surface high-magnification SEM of the black bead (a) In P1, only a small amount of palladium nanoparticles are attached to the surface of the support; (b) In P2, a large number of palladium nanoparticles are attached to the surface of the support, and the pore structure is still clearly visible; (c) In P5, an excessive amount of palladium nanoparticles are attached to the surface of the support, aggregating into large-sized particles, and the pore structure is almost invisible.

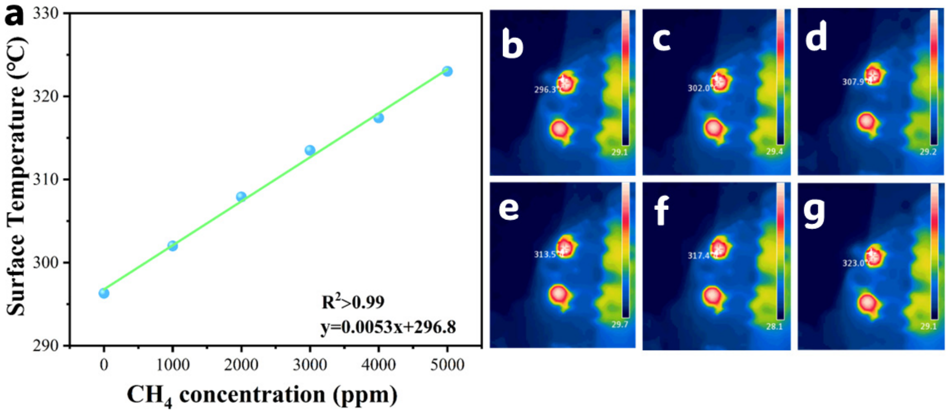

Figure S2. (a) Surface temperature of the black bead at different methane concentrations (0-5000 ppm); (b-g) Thermal imaging results for the black bead at different methane concentrations (0-5000 ppm).
